# The presence of genes encoding enzymes that digest carbohydrates in coral genomes and analysis of their activities

**DOI:** 10.7717/peerj.4087

**Published:** 2017-11-28

**Authors:** Yuki Yoshioka, Toshiaki Tanabe, Akira Iguchi

**Affiliations:** Department of Bioresources Engineering, National Institute of Technology, Okinawa College, Nago-City, Okinawa, Japan

**Keywords:** Cellulase, Coral enzymes, Enzyme activity, Gene expression, Chitinase

## Abstract

Numerous enzymes that digest carbohydrates, such as cellulases and chitinases, are present in various organisms (e.g., termites, nematodes, and so on). Recently, the presence of cellulases and chitinases has been reported in marine organisms such as urchin and bivalves, and their several roles in marine ecosystems have been proposed. In this study, we reported the presence of genes predicted to encode proteins similar to cellulases and chitinases in the genome of the coral *Acropora digitifera*, their gene expression patterns at various life stages, and cellulose- and chitin-degrading enzyme activities in several coral species (*A. digitifera, Galaxea fascicularis, Goniastrea aspera, Montipora digitata, Pavona divaricata, Pocillopora damicornis,* and *Porites australiensis*). Our gene expression analysis demonstrated the expressions of these cellulase- and chitinase-like genes during various life stages, including unfertilized eggs, fertilized eggs, zygotes, planula larvae, primary polyps and adults of *A. digitifera.* Agar plate assays confirmed cellulase and chitinase activities in the tissues extracted from adult branches of several coral species. These results suggested that corals are able to utilize cellulases and chitinases in their life histories.

## Introduction

Carbohydrates are substances essential for life. They are major energy sources, and monosaccharide derivatives constitute sugar chains on cell surfaces. In benthic aquatic organisms, carbohydrate levels have been suggested to reflect food shortage (Rossi et al., 2006). Reef-building corals, which are the major calcifying organisms supporting the biodiversity of coral reef ecosystems (Roberts et al., 2002), are known to secrete mucus, which is mainly composed of carbohydrates (Wild et al., 2010) and is an important primary product in coral reefs (Wild et al., 2004). Corals are thus considered to utilize carbohydrates for maintaining their life histories and also for high-degree use in material circulation in coral reef ecosystems. However, how corals use carbohydrates is not yet known.

Corals maintain holobiotic relationships with symbiotic algae (zooxanthellae) as well as with bacteria, archaea, and viruses (Rohwer et al., 2002). Recently, cellulase activity was reported in microorganisms related to corals (Sousa et al., 2016). In parallel, available genomic information on corals has greatly increased, and the whole genome sequence of *Acropora digitifera* was reported (Shinzato et al., 2011). Although we confirmed the presence of several genes predicted to encode enzymes that can digest carbohydrates, such as cellulases and chitinases (details are provided below) in this genome, the details of these enzymes have not yet been evaluated.

Cellulose, which consists of D-glucose units with β-1,4-linkages, serves as the main constituent of plant cell walls, and is the most abundant carbohydrate on earth (Watanabe & Tokuda, 2001). Cellulases (e.g., endo-β-1,4-glucanases (EC 3.2.14), cellobiohydrolase (EC 3.2.1.91), and β-glucosidase (EC 3.2.1.21)), which hydrolyse internal β-1,4-linkages in cellulose chains, are widely distributed in various organisms, including plants (Brummell, Lashbrook & Bennett, 1994), fungi (Tomme, Warren & Gilkes, 1995), bacteria (Tomme, Warren & Gilkes, 1995), and molluscs (Watanabe & Tokuda, 2001). Cellulases were initially identified as products of symbiotic or contaminating microorganisms in the digestive tracts of animals (Martin & Martin, 1978). However, recent studies have shown that cellulases are present as products of the hosts themselves in terrestrial animals, such as termites (Watanabe et al., 1998), nematodes (Smant et al., 1998), and beetles (Sugimura et al., 2003), and also in marine organisms such as abalones (Suzuki, Ojima & Nishita, 2003), sea urchins (Nishida et al., 2007), and bivalves (Sakamoto et al., 2007). Chitin, which consists of repeating N-acetyl-β-D-glucosamine units with β-1,4-linkages, is the main component of cuticular exoskeleton (Merzendorfer & Zimoch, 2003) and is as abundant as cellulose (Merzendorfer & Zimoch, 2003). Chitinases (EC 3.2.1.14), which randomly cleave hydrogen β-1,4-linkages in chitin and provide protection against bacterial invasion (Merzendorfer & Zimoch, 2003), is similarly widely distributed in organisms such as insects (Merzendorfer & Zimoch, 2003), hydroids (Klug et al., 1984), and fish (Matsumiya & Mochizuki, 1996; Ikeda et al., 2014; Kawashima et al., 2016). Cellulases and chitinases in corals are likely to play roles in carbohydrate utilization; however, there is virtually no information on these enzymes in corals.

This study was aimed at examining the fundamental aspects of the genes encoding enzymes that digest carbohydrates, such as cellulase and chitinase. To analyse the gene expression of these enzymes, we examined the gene expression patterns at various life stages of *A. digitifera* using the DNA sequences of enzymes from genomic information of this species. Additionally, the cellulase and chitinase activities were evaluated by agar plate assays using protein samples from seven dominant coral species: *A. digitifera, Galaxea fascicularis, Goniastrea aspera, Montipora digitata, Pavona divaricata, Pocillopora damicornis,* and *Porites australiensis*. Based on these results, we discussed the possible functions of cellulases and chitinases in corals and future directions for studies on genes encoding carbohydrate-catabolising enzymes in corals.

## Materials and Methods

### Sample collection

Gametes were collected from five *A. digitifera* colonies that spawned in May 2015. Then, samples at various life stages were collected and frozen at −80 °C until use. Fragments of adult corals of seven species, namely *A. digitifera, G. fascicularis, G. aspera, M. digitata, P. divaricata, P. damicornis, and P. australiensis*, were collected from the fringing reef of Sesoko Island, Okinawa, Japan. All samplings were conducted with permission from Okinawa Prefecture, Japan (permission No. 28-4). The collected fragments were maintained in a tank with running seawater under natural light conditions at Sesoko Station, Tropical Biosphere Research Centre, University of the Ryukyus, Okinawa, Japan, until use.

### Search and design of primers for cellulase- and chitinase-likes genes in coral genome

We searched the genome database of *A. digitifera* (v1.1) (http://marinegenomics.oist.jp/acropora_digitifera) for cellulases and chitinases. The sequences of coral cellulase-like genes were obtained from predicted *A. digitifera* transcripts through BLASTP analysis (Camacho et al., 2009) (Table 1) using the sequence of *Corbicula japonica* cellulase (Accession No. AB264777). The sequences of *A. digitifera* predicted transcripts were annotated against the Swiss-Prot (http://www.uniprot.org). Based on the annotated information, we obtained chitinase-like genes of *A. digitifera*. PCR primers for each gene, which were designed to amplify 100–200-bp fragments and spanned the exon-intron boundaries inferred from the GTF file of *A. digitifera* converted from the GFF file (v1.1) using Cufflinks 2.2.1 (Trapnell et al., 2010), were designed using Primer3Plus (Untergasser et al., 2007) (Table 2). The PCR primers were designed to amplify virtually full-length cDNAs based on predicted *A. digitifera* transcripts (v1.0) (Table S1).

**Table 1:** BLAST analysis of cellulase-like genes in coral genome using sequence of *Corbicula japonica* cellulase (Accession No. AB264777).

Target gene	Gene ID	Score	*E*-value
Cellulase-like-1	adi_v1.03986	206	6.00E−60
Cellulase-like-2	adi_v1.23389	204	1.00E−59

**Table 2:** RT-PCR primers for cellulase- and chitinase-like genes. GAPDH served as the internal control. Gene IDs of predicted transcripts of target genes of *Acropora digitifera* (v1.1) are enclosed within parentheses.

Target gene	Forward primer (5′-3′)	Reverse primer (5′-3′)	Product size
Cellulase-like-1 (aug_v2a.03986)	CCACAGACTACCTCATTAAA	ATCGTCATATTTTCTGGTCT	109 bp
Cellulase-like-2 (aug_v2a.23389)	CCACAGACTACCTCATTAAA	GAACCATCGTCATATTTTC	114 bp
Chitinase-like-1 (aug_v2a.00687)	TCAGTGTGTGACAATTTTAG	TATATATCATTCGCTTGTCA	111 bp
Chitinase-like-2 (aug_v2a.09189)	ATTTAACAAACATGACTTCG	CTTGTCCGTAAAAGTTAAGA	197 bp
GAPDH (aug_v2a.06879)	ATTGGAAGGCTGGTTAT	CGTCTTTCACCTCTGTAGT	154 bp

**Figure 1:**
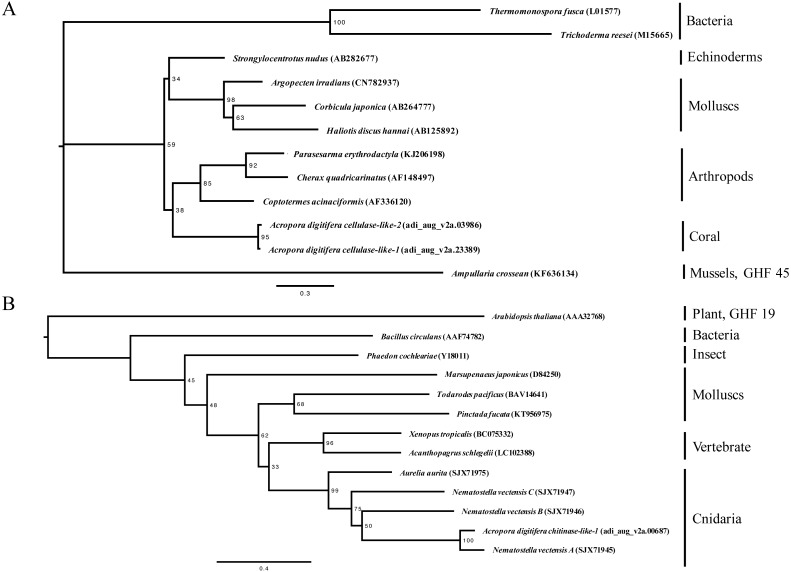
Maximum-likelihood trees (1,000 bootstraps samples) for (A) cellulases (the WAG + G evolution model was selected) and (B) chitinases (the WAG + G + I evolution model was selected). Numbers at the branch points indicate the percentage of 1,000 bootstrap replicates supporting the topology. GenBank IDs are provided within parentheses. The bar represents 0.2 amino acid substitutions per site.

### Gene expression analysis by reverse transcription-polymerase chain reaction (RT-PCR)

Total RNA was isolated from tissue samples of *A. digitifera* at different life stages (unfertilized eggs, fertilized eggs, zygotes after 15 h after fertilization, zygotes after 40 h after fertilization, planula larvae, primary polyps, and adult branches) with TRIzol solution (Invitrogen), and the isolated RNA samples (900 ng) were reverse-transcribed to cDNA using the PrimeScript RT reagent kit with gDNA Eraser (Takara). RT-PCR was conducted as follows: denaturation at 94 °C for 1 min, followed by 35 cycles of denaturation at 94 °C for 30 s, annealing at 55 °C for 30 s, and extension at 72 °C for 2 min, with a final extension step at 72 °C for 5 min. The reactions were performed in a thermal cycler (Veriti; Applied Biosystems, Foster City, CA, USA) and each 10-µL reaction mixture contained 1 µL of each primer at 5 µM, 1 µL of template cDNA, 2 µL of UltraPure DNase/RNase Free-distilled water (Thermo Fisher Scientific, Waltham, MA, USA), and 5 µL of Premix Ex Taq (TaKaRa, Shiga, Japan). Then, 4 µL of each PCR product was subjected to 2% agarose gel electrophoresis to confirm amplification as a single band visualized with GelRed (Wako, Osaka, Japan) under ultraviolet light.

### Agar plate assays of cellulase and chitinase activities

Branches from adult corals of seven species (*A. digitifera, G. fascicularis, G. aspera, M. digitata, P. divaricata, P. damicornis, and P. australiensis*) were crushed using an iron mortar and pestle on ice and homogenized using stainless steel beads in PBS buffer (pH 7.4) at 3,000 rpm for 5 s. The crushed coral samples were centrifuged at 10,000 g for 10 min at 4 °C and the supernatants were concentrated using a disposable ultrafiltration unit (type USY-1; Advantec, Dublin, CA, USA). The protein concentration in the supernatants was adjusted to 2 mg/mL, and then, the supernatants were used as enzyme solutions. Agarose plates containing 1.5% agarose, acetate buffer (pH 5.5), and 0.1% carboxymethylcellulose (CMC) (Sigma) were prepared for assessing cellulase activity in the corals. To assess chitinase activity in the corals, agarose plates containing 0.1% glycol chitin were prepared according to the method of Yamada & Imoto (1981). In brief, 5 µL of enzyme solution was deposited on the centre of each plate and the plate was incubated at 25 °C for 48 h. Then, the plates were stained with 0.1% Congo red (Wako) for 1 h and de-stained with 1 M NaCl solution.

### Phylogenetic analysis

The amino acid sequences of the cellulase- and chitinase-like genes of *A. digitifera* were used for phylogenetic analysis. Representative protein sequences of cellulase and chitinase in other organisms were selected from GenBank. The amino acid sequences were aligned using Mafft v7.309 (Katoh & Standley, 2013). Chitinase-like-2 gene (aug_v2a.09189) was excluded from the phylogenetic analysis because of the short length of its sequence compared with the others. Then, the best-fit evolution model was selected using the Akaike Information Criterion (AIC) with ProtTest 3.4.2 (Darriba et al., 2011) and the maximum-likelihood trees were constructed by RAxML v8.2.10 (Stamatakis, 2014) with analysis of 1,000 bootstrap samples, and visualized with FigTree v1.4.3 (http://tree.bio.ed.ac.uk/software/figtree/).

**Figure 2:**
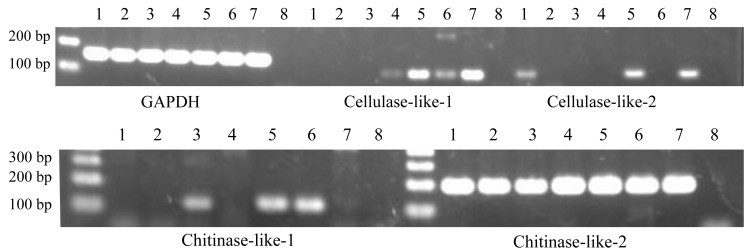
Expression patterns of cellulase- and chitinase-like genes in certain life stages of *Acropora digitifera*. 1, Unfertilized eggs; 2, Fertilized eggs; 3, Zygotes 15 h after fertilization; 4, Zygotes 40 h after fertilization; 5, Planula larvae; 6, Primary polyps; 7, Adult branch; 8, Negative control. GAPDH served as the internal control.

## Results and Discussion

In this study, we identified two cellulase-like genes and two chitinase-like genes in the *A. digitifera* genome (Table 1). Phylogenetic analysis supported that these cellulase- and chitinase-like genes were classified into the animal-origin group (Fig. 1). Regarding the cellulase-like genes, two coral cellulase-like genes belonged to the GHF 9 group and were located adjacent to each other owing to high sequence similarity at the putative amino acid level. Regarding the chitinases, two types, namely, animal and plant, are known and a coral chitinase-like gene was classified into the GHF 18 group in the phylogenetic tree.

Similarly, we confirmed their gene expression at the various life stages of *A. digitifera*. Regarding RT-PCR with partial sequences, the cellulase-like-1 gene was expressed at four stages (40 h after fertilization, planula larvae, primary polyp, and adult branches; Fig. 2), cellulase-like-2 gene at three stages (unfertilized eggs, planula larvae, and adult branches; Fig. 2), chitinase-like-1 gene at three stages (15 h after fertilization, planula larvae, and primary polyp; Fig. 2), and chitinase-like-2 gene at all the analysed stages (Fig. 2). Regarding RT-PCR with longer sequences, the cellulase-like-1 gene was expressed in the planula larvae and adult branches (Fig. S1) and the chitinase-like-2 gene at all the analysed stages (Fig. S1); however, the expression of the cellulase-like-2 and chitinase-like-1 genes was not confirmed. These results suggested that these genes perform certain functional roles in corals and the differences in the gene expression patterns indicate that these genes may have different roles.

**Figure 3:**
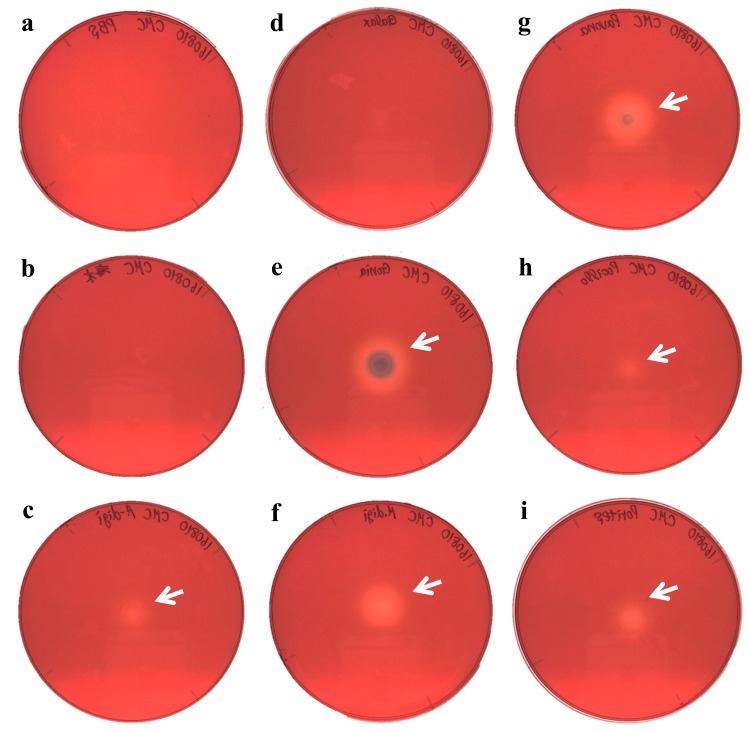
Agar plate assays for cellulase. (A) Negative control (PBS); (B) Negative control (Sea water around coral samples); (C) *Acropora digitifera*; (D) *Galaxea fascicularis*; (E) *Goniastrea aspera*; (F) *Montipora digitata*; (G) *Pavona divaricata*; (H) *Pocillopora damicornis*; (I) *Porites australiensis*. White arrows indicate the site on substrate degraded by the dropped enzyme solution.

**Figure 4:**
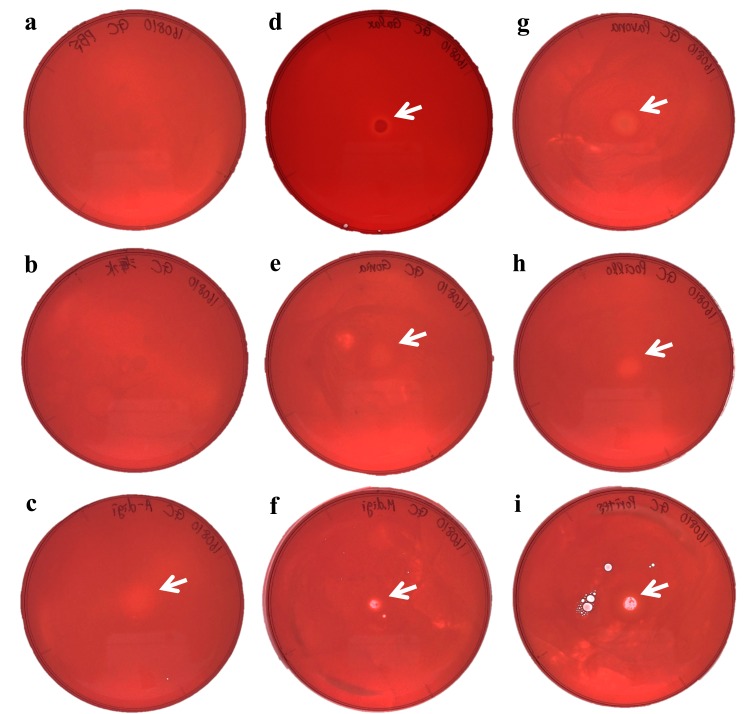
Agar plate assays for chitinase. (A) Negative control (PBS); (B) Negative control (Sea water around coral samples); (C) *Acropora digitifera*; (D) *Galaxea fascicularis*; (E) *Goniastrea aspera*; (F) *Montipora digitata*; (G) *Pavona divaricata*; (H) *Pocillopora damicornis*; (I) *Porites australiensis*. White arrows indicate the site on substrate degraded by the dropped enzyme solution.

We demonstrated cellulase activity in six of seven coral species (*A. digitifera, G. aspera, M. digitata, P. divaricata, P. damicornis, and P. australiensis*; Fig. 3) and chitinase activity in all the seven coral species (Fig. 4), suggesting the presence of cellulases and chitinases in a wide range of coral taxa. We observed that the host coral genome contained genes predicted to encode cellulase- and chitinase-like enzymes and these genes were expressed at various life stages. Thus, the enzyme activities observed in this study may be attributed to the coral hosts. Meanwhile, corals are known to be holobionts of coral hosts, symbiotic *Symbiodinium* spp., and coral-related bacteria (Bourne, Morrow & Webster, 2016; Rohwer et al., 2002). The cellulase and chitinase activities observed in this study could be attributed to the bacteria surrounding the corals. Thus, to clarify the origin of the enzyme activities demonstrated in this study, we should perform further functional analyses of cellulase- and chitinase-like genes in coral genomes and DNA barcode analyses targeting bacteria may be helpful in future studies.

Corals may be able to utilize cellulases and chitinases to assimilate carbohydrates obtained by digesting plankton and detritus from the surrounding environment (Bourne, Morrow & Webster, 2016). Corals are known to be capable of heterotrophic nutrition to supplement their energy budget (Grottoli, Rodrigues & Palardy, 2006) and consumption of zooplankton, such as copepods, with chitinous exoskeletons that could be digested by chitinases. In corals, chitin is reported to be used in synthesising skeletons (Watanabe et al., 2003), suggesting the contribution of chitin to physiological functions in corals. Thus, chitinases would be indispensable for obtaining chitin to maintain corals life histories. The role of cellulase in corals may be closely related to the presence of symbiotic algae. Harii et al. (2009) reported that *A. digitifera* can acquire symbiotic zooxanthellae at the planula larval stage. Interestingly, our results showed that the cellulase-like genes were mainly expressed after the planula larval stage (Fig. 2, Fig. S1). The cell wall of *Symbiodinium* spp. was reported to be composed of a stable shell of cellulose (Markell, Trench & Iglesiasprieto, 1992). Additionally, the degradation of zooxanthellae was reported to be caused by the digestion of symbionts by the coral host (Titlyanov et al., 1996; Titlyanov et al., 1998). Thus, corals might digest zooxanthellae with cellulase to establish better symbiotic relationships. Furthermore, the degree of dependence of corals on zooxanthellae is suggested to be different among species (Grottoli, Rodrigues & Palardy, 2006), which may be related to the deficiency in cellulase activity in *Galaxea fascicularis* observed in this study.

There may be other possible roles for these coral cellulase- and chitinase-like enzymes. Chitinase has been shown to play a role in defense against fungal pathogens (Kramer & Muthukrishnan, 1997; Ng, 2004). Douglas et al. (2007) suggested that chitinase in sea fan corals is used in defence against chitinaceous pathogens and released into the surrounding seawater. Moreover, the mucus of the coral *Acropora palmata* was reported to have antibiotic properties (Ritchie, 2006). Thus, corals may utilize chitinases in defence against invading organisms. Coral reef ecosystems have been degraded by anthropogenic activities (e.g., nutrient pollution and overfishing). Under these conditions, coral reefs are expected to undergo substantial changes from healthy states to algal-dominant states (Bellwood et al., 2004). Thus, to counteract surrounding invasive algae, coral might produce cellulase-like enzymes to protect themselves from potentially invasive algae by digestion with cellulose (Tanaka et al., 2013). Further studies are required to clarify the roles of the cellulase- and chitinase-like enzymes described in this study.

##  Supplemental Information

10.7717/peerj.4087/supp-1 Figure S1

10.7717/peerj.4087/supp-2 Table S1

## Additional Information and Declarations

### Field Study Permissions

The following information was supplied relating to field study approvals (i.e., approving body and any reference numbers): All samplings were carried out after obtaining permission from Okinawa Prefecture, Japan.

### Data Availability

The following information was supplied regarding data availability: The raw code is included in the ‘Materials and Methods’ section of the manuscript.
