# Supplementary figures and images for: The presence of genes encoding enzymes that digest carbohydrates in coral genomes and analysis of their activities
Source: PeerJ. 2017 Nov 28;5:e4087. doi: 10.7717/peerj.4087 (PMC5710165; doi:10.7717/peerj.4087)

1 2 3 4 5 6 7 8

GAPDH

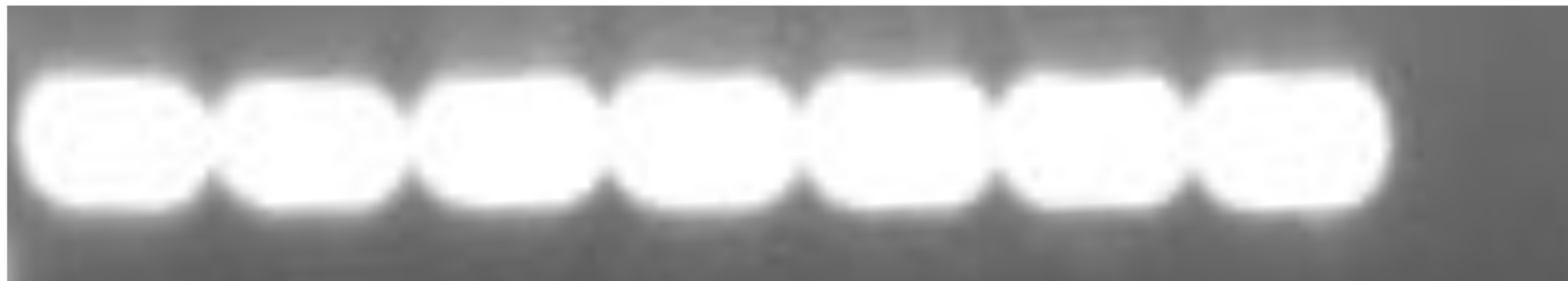

Cellulase-like-1

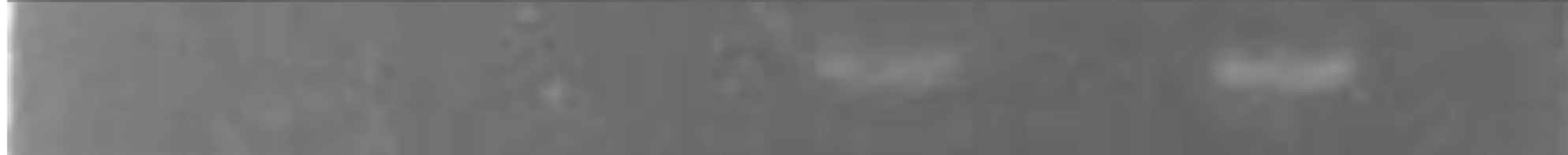

Chitinase-like-2

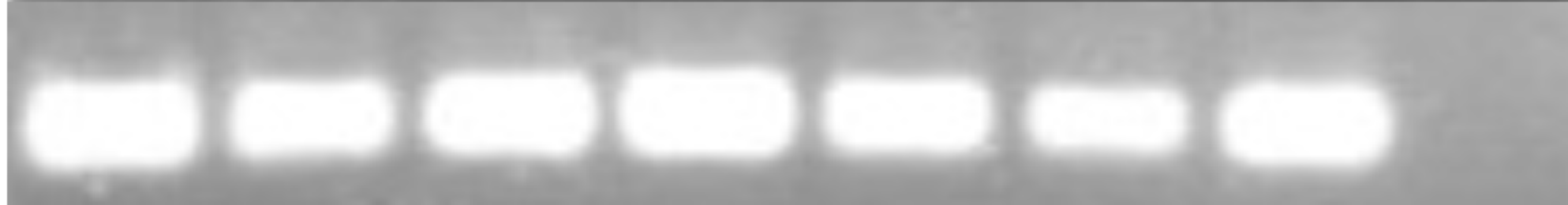

Supplement: Figure S1 [file peerj-05-4087-s001.pdf]
