# Supplementary material for: The presence of genes encoding enzymes that digest carbohydrates in coral genomes and analysis of their activities
Source: PeerJ. 2017 Nov 28;5:e4087. doi: 10.7717/peerj.4087 (PMC5710165; doi:10.7717/peerj.4087)
Supplement: Table S1 [file peerj-05-4087-s002.docx]

**Table S1** RT-PCR primers for cellulase- and chitinase-like genes. GAPDH served as the internal control. Gene IDs of predicted transcripts of target genes of *Acropora digitifera* (v1.0) are enclosed within parentheses.

|  |  |  |  |
| --- | --- | --- | --- |
| Target gene | Forward Primer (5'-3') | Reverse primer (5'-3') | Product size |
| Cellulase-like-1 (adi_v1.03986) | CCCTGTCGCTGTTCTGAATC | GGTCGAGCCATCGTCATATT | 831 bp |
| Chitinase-like-2 (adi_v1.09189) | CTTGTATCTTCTTGTCAGCAAGC | CGATATATCTTGATCCCGTGATAG | 1400 bp |
|  |  |  |  |
